# Overcoming barriers for left atrial appendage thrombus: a systematic review of left atrial appendage closure

**DOI:** 10.1186/s12872-024-03843-w

**Published:** 2024-03-21

**Authors:** Zixi Zhang, Jiabao Zhou, Qiuzhen Lin, Cancan Wang, Yunying Huang, Yongguo Dai, Wanyun Zuo, Na Liu, Yichao Xiao, Qiming Liu

**Affiliations:** 1 Department of Cardiovascular Medicine, The Second Xiangya Hospital, Central South University, 139 Renmin Road, Furong District, Hunan Province, Changsha, 410011 People’s Republic of China; 2 Department of Metabolic Endocrinology, The Second Xiangya Hospital, Central South University, Hunan Province, Changsha, 410011 People’s Republic of China; 3 Department of Pharmacology, Wuhan University TaiKang Medical School (School of Basic Medical Sciences), Hubei Province, Wuhan, 430071 People’s Republic of China; 4 Department of Hematology, The Second Xiangya Hospital, Central South University, Hunan Province, Changsha, 410011 People’s Republic of China

**Keywords:** Atrial fibrillation, Left atrial appendage thrombus, Left atrial appendage closure, Systematic review

## Abstract

**Background:**

Approximately 90% of intracardial thrombi originate from the left atrial appendage in non-valvular atrial fibrillation patients. Even with anticoagulant therapy, left atrial appendage thrombus (LAAT) still occurs in 8% of patients. While left atrial appendage closure (LAAC) could be a promising alternative, the current consensus considers LAAT a contraindication to LAAC. However, the feasibility and safety of LAAC in patients with LAAT have yet to be determined.

**Methods:**

This systematic review synthesizes published data to explore the feasibility and safety of LAAC for patients with LAAT.

**Results:**

This study included a total of 136 patients with LAATs who underwent successful LAAC. The Amulet Amplatzer device was the most frequently utilized device (48.5%). Among these patients, 77 (56.6%) had absolute contraindications to anticoagulation therapy. Cerebral protection devices were utilized by 47 patients (34.6%). Transesophageal echocardiography (TEE) is the primary imaging technique used during the procedure. Warfarin and novel oral anticoagulants were the main anticoagulant medications used prior to the procedure, while dual antiplatelet therapy was primarily used post-procedure. During a mean follow-up period of 13.2 ± 11.5 months, there was 1 case of fatality, 1 case of stroke, 3 major bleeding events, 3 instances of device-related thrombus, and 8 cases of peri-device leakage.

**Conclusions:**

This review highlights the preliminary effectiveness and safety of the LAAC procedure in patients with persistent LAAT. Future large-scale RCTs with varied LAAT characteristics and LAAC device types are essential for evidence-based decision-making in clinical practice.

**Supplementary Information:**

The online version contains supplementary material available at 10.1186/s12872-024-03843-w.

## Introduction

Atrial fibrillation (AF) is a rapid supraventricular arrhythmia characterized by irregular electrical activity and ineffective atrial contractions. The incidence of AF gradually increases with age and has emerged as a significant public health concern [1]. Stroke, as one of the severe complications of AF, often results in cardioembolic events that are not only severe but also have a high risk of recurrence. These strokes are frequently fatal or lead to permanent disability [2]. AF is associated with a 4- to fivefold increased risk of ischemic stroke and accounts for 25% of the 700 000 cerebrovascular accidents that occur in the United States annually [3]. Finding effective strategies to mitigate the risk of stroke associated with AF has become a crucial concern for cardiovascular physicians.

In patients with nonvalvular AF, approximately 90% of intracardiac thrombi originate from the left atrial appendage (LAA). Existing evidence indicates that the majority of strokes in patients with AF result from embolization of the left atrial appendage thrombus (LAAT) [4]. Oral anticoagulant (OAC) therapy is currently the primary approach for preventing and treating thrombosis associated with AF [5]. Despite anticoagulant treatment, LAAT still occurs in 8% of patients [6]. Moreover, a comprehensive assessment of the risk of drug-related bleeding is crucial for patients with renal dysfunction or active bleeding before initiating anticoagulant therapy. These factors limit the utilization of OACs.

For patients contraindicated for OAC therapy, a dilemma arises owing to the increased bleeding risk associated with OAC therapy and the consistently high risk of thrombosis resulting from LAAT. In such cases, the left atrial appendage closure (LAAC) procedure has emerged as a promising alternative. Recent clinical trials have established that LAAC procedure is not only noninferior to OAC therapy in terms of preventing thromboembolic events but also offers a significant reduction in bleeding complications. Furthermore, it is important to note that studies, such as the one published in JAMA 2014, have demonstrated a survival benefit for LAAC patients. This benefit, which was initially observed to be non-significant, became increasingly pronounced over the years, providing compelling evidence for the long-term efficacy of LAAC procedure over OAC therapy in certain patient populations [7, 8]. Previously, the presence of LAAT was considered a contraindication for LAAC, and landmark clinical trials on LAAC did not include this specific patient population [9–11]. The effectiveness and safety of LAAC in patients with LAAT have not been validated. However, recent studies have revealed that LAAC can effectively prevent stroke events in patients with LAAT, with minimal procedural complications [12, 13]. These findings suggest that LAAT may not be an absolute contraindication for LAAC. Currently, there are limited available data on the use of LAAC for patients with LAATs [14]. This systematic review compiles the most recent relevant studies to explore the feasibility and safety of LAAC in patients with LAAT.

## Methods

### Search strategy

This systematic review examined the available data on AF patients who underwent LAAT and LAAC procedures. A comprehensive search covering the period from January 1, 2000, to June 1, 2023, was conducted in the PubMed, EMBASE, Google Scholar, and SpringerLink databases to identify all relevant abstracts or full-text cases of LAAC in patients with LAAT. The search terms "left atrial appendage occlusion" OR "LAAO" OR "left atrial appendage closure" OR "LAAC" OR "Watchman" OR "Watchman-Flex" OR "Amplatzer cardiac plug" OR "ACP" OR "Amulet" and "left atrial appendage thrombus" were used to identify relevant articles. A manual search of the selected articles and relevant references in published reviews was performed to ensure comprehensive data collection.

### Data extraction and statistical analysis

**Fig. 1:**
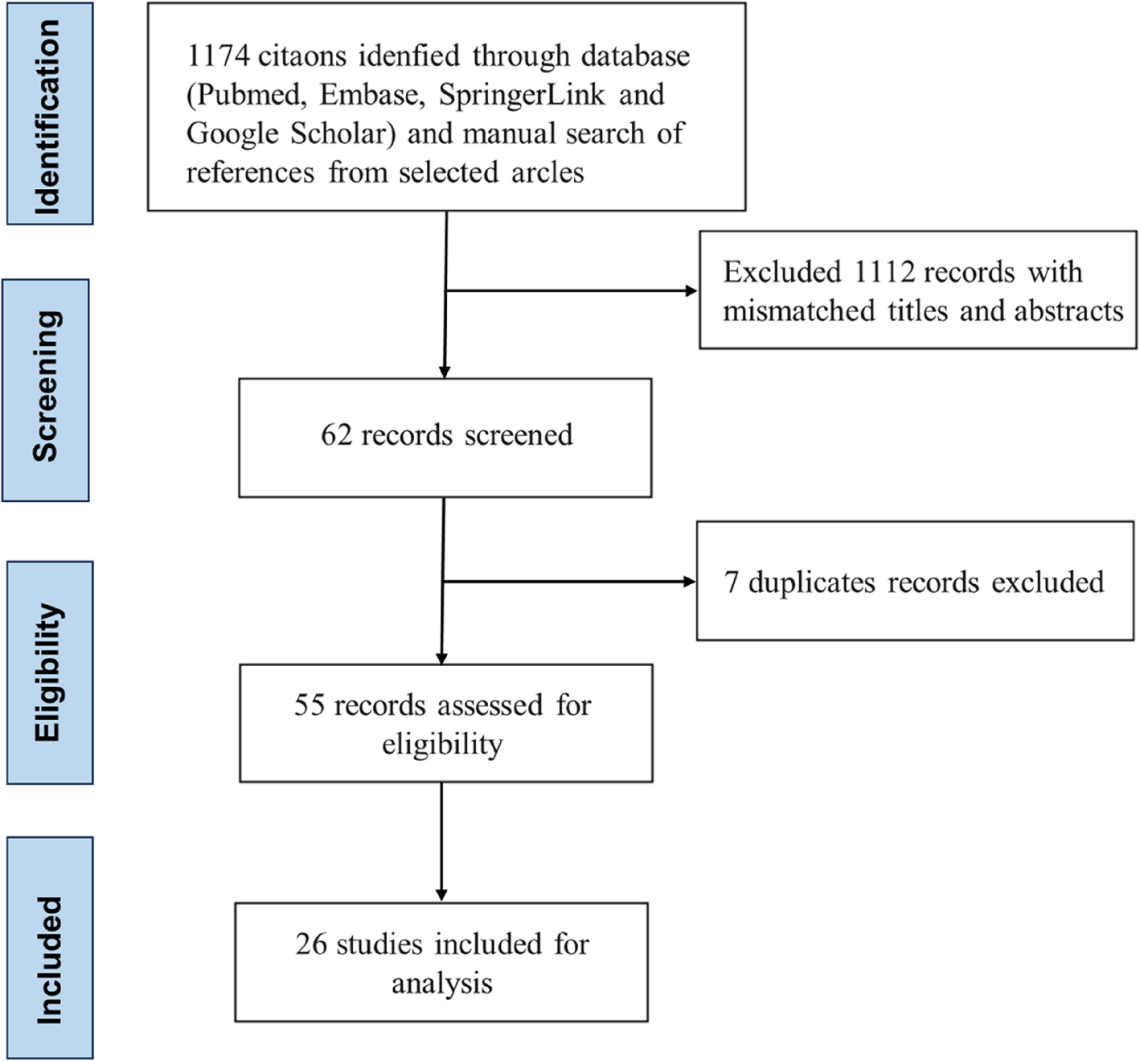
Study selection flowchart

Studies and abstracts reporting cases of LAAC device placement in patients with LAAT were included in our analysis. We excluded articles lacking detailed procedures and essential follow-up data. Non-English language articles were also excluded. Clinical follow-up data, including thromboembolic events, major bleeding events, device-related thrombus (DRT), peri-device leakage (PDL), complications related to the closure device, transient ischemic attack, death and the use of anticoagulation or antiplatelet therapies, were collected from each study. Following a successful LAAC procedure, the physician decided to discontinue anticoagulation therapy and switch to antiplatelet agents. Continuous data are expressed as the mean ± standard deviation (M ± SD) for normally distributed variables or as the median (25th, 75th percentiles) for non-normally distributed variables. A statistical analysis using IBM SPSS version 26 was performed to analyze the pooled data. The study selection process is represented in Fig. 1.

### Definition of terms

Device success was defined as the deployment of the occluder in the correct position with < 5 mm of a PDL. Adverse clinical outcomes included a composite of death, stroke or other systemic thromboembolism, DRT, major bleeding, PDL, transient ischemic attack and device-related complications during follow-up. Major bleeding events included gastrointestinal bleeding, cerebral hemorrhage, intramuscular bleeding, or active bleeding directly related to the OAC therapy. PDL refers to postprocedural leakage exceeding 5 mm. A DRT was defined as a thrombus that formed on the LAAC device. Anticoagulation therapy after the procedure includes warfarin or novel oral anticoagulants (NOACs).

### Types of LAAC devices

The commonly used LAAC devices include Watchman, Watchman FLX, Amplatzer Cardiac Plug (ACP), Amulet Amplatzer, and Lambre devices. The Watchman device (Boston Scientific, MA) is the most extensively studied percutaneous LAAC device. It consists of a self-expanding nitinol frame with fixation anchors and is covered with a polyethylene terephthalate fabric membrane on the proximal face [15]. The implantation procedure typically involves a standard transseptal puncture after femoral vein access. Subsequently, the 14F Watchman Access Sheath is exchanged using a guidewire, and the pigtail catheter is used to deliver the device into the LAA. The release of the device must meet the "PASS" criteria [16].

The ACP is a dual-disc LAAC device that structurally resembles the Amplatzer atrial septal occluder. The distal disc is placed in the LAA to prevent displacement, while the proximal disc cap seals the orifice of the LAA. The Amulet Amplatzer device is a second-generation LAAC device based on the ACP design. Compared to the ACP, the Amulet Amplatzer device incorporates additional anchoring hooks, a deeper distal lobe, a longer waist, and a recessed distal screw to minimize exposed metal within the LAA and subsequently reduce the incidence of DRT [17].

Lifetech received CE Mark approval for the LAmbre closure system on 15 June 2016. The closure system has a double umbrella design with two layers of polyethylene terephthalate fabric in the cover and umbrella. The implant is a nitinol-based, self-expanding device comprising a hook-embedded umbrella with a short central waist. The waist acts as an articulating, compliant connection between the cover and the umbrella, allowing the cover to self-orient the cardiac wall [18].

## Results

### Baseline characteristics

**Fig. 2:**
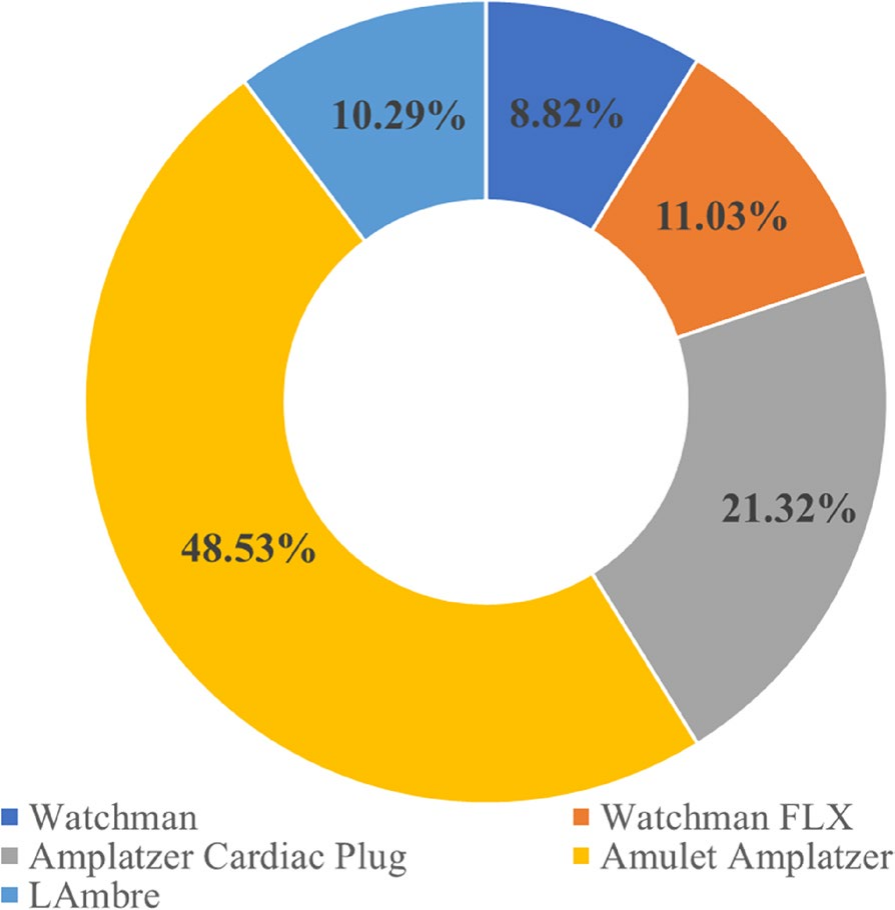
Usage distribution of different LAAC devices. LAAC, left atrial appendage closure

We conducted a comprehensive analysis of 136 patients from 26 publications. The median age was 64 years, and 86 (63.2%) of the patients were male. The most common comorbidities observed were hypertension (36%) and diabetes (36.8%). Permanent AF accounted for approximately 53.7% of the patients. A total of 47 patients (34.6%) received implantation of a cerebral protection device (CPD). The most frequently used device was the Amulet Amplatzer (48.5%), and Fig. 2 provides an overview of the types of LAAC devices used. Among the patients, 43.3% had absolute contraindications to anticoagulation therapy due to the risk of major bleeding, while 56.7% of patients had relative contraindications due to experienced thromboembolic events despite receiving OAC treatment or declining to adhere to anticoagulant medication. The detailed baseline characteristics of the patients are presented in Table 1.

**Table 1:** Baseline characteristics of the patients

Age, years	70.5 (67–76)
Male, (%)	86 (63.2)
Previous stroke, (%)	53 (39.0)
Previous bleeding, (%)	64 (47.1)
Hypertension, (%)	49 (36.0)
Congenital heart disease, (%)	3 (2.2)
Diabetes mellitus, (%)	50 (36.8)
Cardiomyopathy, (%)	1 (0.7)
Heart failure, (%)	26 (19.1)
CHA_2_DS_2_-VASc score	4.4 (3.6–6)
HAS-BLED score	3 (3–4)
LAA electrical isolation, (%)	17 (12.5)
Electrical cardioversion, (%)	11 (8.1)
Embolic protection device, (%)	47 (34.6)
Types of AF	
Paroxysmal AF, (%)	20 (14.7)
Persistent AF, (%)	43 (31.6)
Permanent AF, (%)	73 (53.7)
Types of LAAC devices	
Watchman, (%)	12 (8.8)
Watchman FLX, (%)	15 (11.0)
ACP, (%)	29 (21.3)
Amulet Amplatzer, (%)	66 (48.5)
LAmbre, (%)	14 (10.3)

*ACP* Amplatzer cardiac plug, *AF* atrial fibrillation, CHA_2_DS_2_-VASc, congestive heart failure, hypertension, age ≥ 75 years, diabetes mellitus, stroke, vascular disease, age 65–74 years, sex category, HAS-BLED hypertension, abnormal renal/liver function, stroke, bleeding history or predisposition, labile international normalized ratio, elderly, drugs/alcohol concomitantly, *LAA* left atrial appendage, *LAAC* left atrial appendage closure

Continuous data are summarized as *n* (%) or median (interquartile range)

### Antiplatelet and anticoagulation therapy

**Fig. 3:**
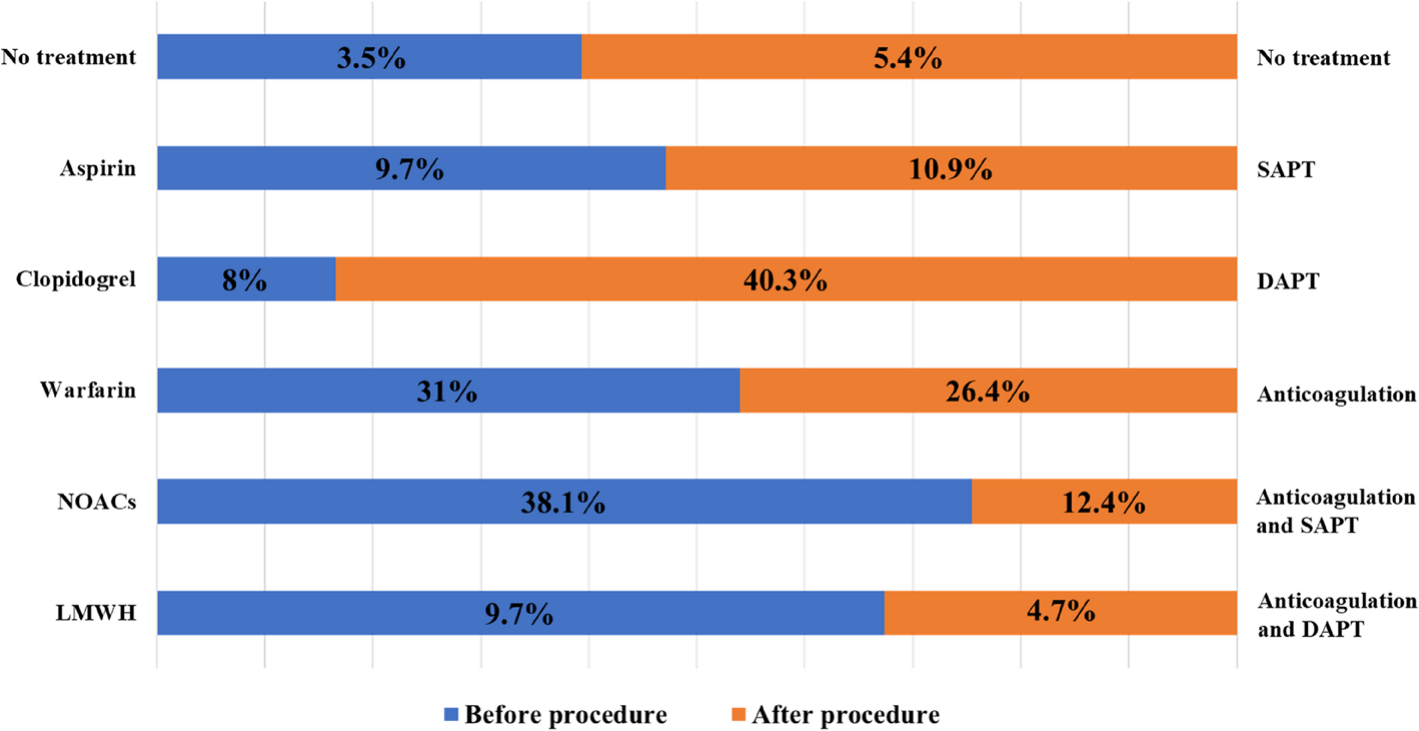
Regimens for antiplatelet and anticoagulation therapy before and after the LAAC procedure**.** DAPT, dual antiplatelet therapy; LAAC, left atrial appendage closure; LMWH, low-molecular-weight heparin; NOACs, novel oral anticoagulants; SAPT, single antiplatelet therapy

Comparing the preprocedural and postprocedural antiplatelet and anticoagulation strategies for LAAC, it was noted that NOACs (38.1%) and warfarin (31%) were the primary OACs used before the procedure. Although the current guidelines do not recommend the use of antiplatelet drugs alone for the prevention or treatment of AF-related embolism, some patients still receive aspirin or clopidogrel as their OAC strategy. Dual antiplatelet therapy (DAPT) emerged as the most common postprocedural treatment approach and accounted for 40.3% of the patients. Detailed information about antiplatelet and anticoagulation therapy is presented in Fig. 3.

### Procedural characteristics

In all patients, the occluder was successfully positioned in the correct location. While the majority of procedures adhered to a standard protocol, modifications were made in some cases to enhance safety, particularly in the presence of LAAT. These included the utilization of the 'no-touch technique', which is especially critical in avoiding manipulation within the LAA using any sheath, catheter, or guidewire. It is also important to note that the customary practice of employing a pigtail catheter for local injection in LAAC is contraindicated in these scenarios. This is due to the risks posed by retracting the pigtail catheter before the introduction of the LAAC device. Detailed descriptions of these procedural modifications are provided in Table 2.

**Table 2:** Procedural characteristics

Study	Device	LAA electrical isolation	Cardioversion	CPD	Device success	Procedural imaging	Procedural perform	Reference
Bordignon 2019	Amulet Amplatzer = 9	6	N/A	N/A	9	TEE	Modified Brockenbrough technique and no LAA angiography. Under 60° to 75° TEE view, the device was advanced in the LAA until the proximal edge of the partially opened lobe was in line with the circumflex coronary artery. Under the counterclockwise torque of the sheath, the lobe was then fully opened in the proximal LAA	[12]
Jin 2022	Lambre = 7	N/A	N/A	2	7	TEE	Transseptal access was obtained via the right femoral vein under fluoroscopic guidance, and a guidewire was slowly advanced into the left atrium. Turned the Swartz sheath clockwise to facilitate the advancement of the guidewire into the left superior pulmonary vein. Then, replaced the Swartz sheath with a delivery sheath and then delivered the pigtail catheter in the left superior pulmonary vein and dropped the pigtail catheter to the LAA ostium by slowly pulling the sheath and catheter back	[13]
Marroquin 2022	Watchman = 1 Watchman FLX = 4 ACP = 3 Amulet Amplatzer = 41 Lambre = 4	N/A	N/A	18	53	TEE	Standard deployment techniques and no-touch technique (involves avoiding guidewire or catheter manipulation within the LAA by loading the closure device with the delivery sheath in the left superior pulmonary vein)	[19]
Beneduce 2019	Watchman FLX = 1	N/A	N/A	1	1	3D TEE and fluoroscopy	No-touch technique. After transseptal puncture, the delivery sheath was pulled back from the left upper pulmonary vein over a stiff guidewire. The device was unsheathed to obtain a ball conformation of its closed distal end, advanced toward the LAA ostium, and successfully deployed under TEE and fluoroscopic monitoring	[20]
Lange 2016	ACP = 1	N/A	N/A	N/A	1	TEE and fluoroscopy	No-touch technique. After placing delivery sheath near proximal LAA, slow injection of contrast followed by advancement of partially open Amulet until the diameter of LAAC exceeded midportion LAA diameter	[21]
Saccà 2017	Amulet Amplatzer = 1	N/A	N/A	1	1	TEE and fluoroscopy	Standard endocardial LAAC procedure	[22]
Jalal 2016	ACP = 1 Amulet Amplatzer = 2	N/A	N/A	N/A	3	3D TEE and fluoroscopy	No-touch technique. Transseptal sheath was advanced into LSPV and over-the-wire exchange was performed with delivery sheath. The delivery sheath was pulled back slightly from the vein and was advanced toward LAA ostium without engaging	[23]
Lee 2018	Watchman = 3 ACP = 6 Amulet Amplatzer = 1	N/A	N/A	N/A	10	TEE and fluoroscopy	Delivery sheath was retracted in front of LAA, and the device was carefully pushed into LAA to avoid touching the thrombus in 4 cases with ACP or Amulet. In 3 cases each with ACP and Watchman, the sheath was gently introduced before the thrombus, and devices were deployed	[24]
Popescu 2022	Lambre = 1	N/A	N/A	1	1	TEE and fluoroscopy	Modified Brockenbrough technique and selective PV and LAA angiography. The SL1 sheath was changed over a guidewire with the LAmbre sheath and the device landing zone diameter was measured by TEE and biplane angiography. The corresponding device was loaded on the delivery system and then advanced in the LA through the LAmbre sheath	[25]
Tan 2021	Watchman = 2	N/A	N/A	1	2	TEE	The Sentinel CPS device was placed using standard practices via the right radial artery with the two filters successfully deployed in the brachiocephalic and left common carotid artery. Using a 14 Fr double curve Watchman access sheath, a 30 mm Watchman device was successfully deployed at the ostium	[26]
Tsai 2023	Watchman = 2 Amulet Amplatzer = 8	10	10	9	10	TEE	No-touch technique. Put the wire in the left superior pulmonary vein and introduced the large LAAC sheath into the left superior pulmonary vein. Then, the wire was withdrawn, and the sheath tip was manipulated and guided to the vicinity of the LAA ostium under the TEE guidance. Then, the Amulet lobe was partially deployed here and then slowly moved into the LAA neck or landing zone under the TEE guidance and then the lobe and disk were fully deployed in 8 cases with Amulet. In 2 cases each with Watchman, put the Watchman sheath system in the left superior pulmonary vein, and gently guided and advanced the sheath system into the LAA by counterclockwise rotation	[27]
Bellmann 2017	ACP = 1	N/A	N/A	N/A	1	TEE and fluoroscopy	No LAA angiography and fishball technique (device lobe is partially deployed until a small lobe). Transseptal sheath was advanced into LSPV and over-the-wire exchange was performed with delivery sheath. Partial deployment of the device takes place in the orifice of the LSPV, then delivery sheath is pulled out and device advanced into LAA	[28]
Aytemir 2016	Amulet Amplatzer = 1	N/A	N/A	N/A	1	3D TEE and fluoroscopy	No LAA angiography. The delivery catheter was advanced up to the LAA ostium and the lobe of the device was pushed to obtain a “ballshape” allowing for better TEE visualization of the device position. Under TEE guidance, the lobe of the Amulet was then carefully advanced up to the landing zone, proximal to the LAA thrombus, and deployed at that level followed by deployment of the disc	[29]
Dugo 2016	Amulet Amplatzer = 1	N/A	N/A	N/A	1	TEE	No LAA angiography. Device was advanced into landing zone under TEE	[30]
Lee 2017	ACP = 1	N/A	N/A	N/A	1	TEE and fluoroscopy	No-touch technique. Made a ball with a lobe of the device by retracting the sheath in front of LAA. Sheath with device was pushed cautiously to the landing zone not to touch the LAA thrombus under guidance of transesophageal echocardiography, the lobe was deployed at position of landing zone followed by deployment of the disc	[31]
De Roeck 2019	Amulet Amplatzer = 1	1	1	1	1	TEE	No LAA angiography. The LAA closure device was successfully implanted solely under TEE guidance	[32]
Chang 2023	Watchman FLX = 2	N/A	N/A	2	2	TEE	Device was advanced into landing zone under TEE	[33]
Mohandes 2020	Lambre = 1	N/A	N/A	1	1	TEE	No LAA angiography. A partial umbrella delivery of a LAmbre 24/30 mm was done in front of LAA ostium and the whole system was advanced up to the point immediately before thrombus in LAA superior lobe	[34]
Kaczmarek 2021	Watchman = 2 Watchman FLX = 2 ACP = 13	N/A	N/A	N/A	17	TEE and fluoroscopy	No LAA angiography in 8 cases. 5 cases underwent LAA angiographies with gentle hand contrast injections through pigtail catheters	[35]
Marcon 2023	Watchman FLX = 6	N/A	N/A	6	6	TEE or ICE	“One shot technique” and a stepwise approach based on continuous ICE monitoring (It consists of ICE guided trans-septal puncture and guidewire advancement within the left superior pulmonary vein; exchange with long delivery sheath; trans-septal crossing with the ICE probe, reaching the point allowing the best LAA view (usually at the LSPV ostium); LAA occluder sizing based on landing zone measurement and LAA occluder deploy under ICE monitoring)	[36]
Cruz-Gonzalez 2017	Lambre = 1	N/A	N/A	1	1	TEE and fluoroscopy	Partial deployment of device at the LAA ostium and it was advanced under simultaneous counterclockwise rotation	[37]
Cammalleri 2016	ACP = 1	N/A	N/A	1	1	TEE	Standard endocardial LAAC procedure	[38]
Pak 2013	ACP = 1	N/A	N/A	N/A	1	TEE	Transseptal sheath was advanced into LSPV and over-the-wire exchange was performed with delivery sheath Then, the sheath was gently rotated to LAA direction. With cautious LAA angiography with minimal touching, device was deployed	[39]
Yadav 2017	Watchman = 2	N/A	N/A	N/A	2	TEE	After standard trans-septal puncture, nonselective angiogram of the LAA was performed and shallow intubation of the appendage, the pigtail catheter positioned to enable telescoping of the delivery sheath into the LAA	[40]
Martins 2018	Amulet Amplatzer = 1	N/A	N/A	1	1	ICE	Standard endocardial LAAC procedure	[41]
Del Furia 2017	ACP = 1	N/A	N/A	1	1	3D TEE	Standard endocardial LAAC procedure	[42]

*ACP* Amplatzer cardiac plug, *CPD* cerebral protection device, *ICE* intracardiac echocardiography, *LAA* left atrial appendage, *LAAC* left atrial appendage closure, *LSPV* left superior pulmonary vein, *TEE* transesophageal echocardiography, *3D* three dimensions

Continuous data are summarized as *n* (%). N/A represents unavailable data. Device success was defined as deployment of the occluder in the correct position with < 5 mm of a PDL

### Follow-up and clinical outcomes

The mean follow-up duration was 13.2 ± 11.5 months. Seven studies reported 16 cases (11.8%) of adverse clinical outcomes, and all patients who underwent CPD implantation were free from stroke events during both hospitalization and follow-up. PDL was the most common adverse clinical outcome, occurring in 8 patients (5.9%). Of these, six patients exhibited PDL measurements ≤ 3 mm, while two had measurements exceeding 5 mm. None of these patients underwent a secondary LAAC. DRT was reported in three patients (2.2%), all of whom were successfully managed with OACs. Major bleeding events were also documented in three patients (2.2%). One patient (0.7%), a 54-year-old male with persistent AF and a CHA_2_DS_2_-VAS_C_ score of 3, underwent ACP implantation without the use of a CPD. Subsequently, the patient experienced a stroke during the follow-up. Moreover, one patient (0.7%) died due to the progression of heart failure. The detailed data concerning the follow-up and clinical outcomes are presented in Table 3.

**Table 3:** Follow-up and clinical outcomes

Study	Follow-Up	Adverse Clinical Outcomes	Reference
Bordignon 2019	4.6 mos	1 major bleeding	[12]
Jin 2022	12.8 mos	1 DRT, 3 PDL	[13]
Marroquin 2022	18 mos	1PDL, 1 major bleeding	[19]
Beneduce 2019	1 mo	N/A	[20]
Lange 2016	1.5 mos	N/A	[21]
Saccà 2017	until discharge	N/A	[22]
Jalal 2016	8.6 ± 2 mos	1PDL	[23]
Lee 2018	27.1 ± 20.3 mos	1 stroke, 1 DRT, 2 PDL	[24]
Popescu 2022	3 mos	N/A	[25]
Tan 2021	3.75 mos	1 major bleeding	[26]
Tsai 2023	20.4 mos	N/A	[27]
Bellmann 2017	3 mos	N/A	[28]
Aytemir 2016	48 h	N/A	[29]
Dugo 2016	1.5 mos	N/A	[30]
Lee 2017	4 d	N/A	[31]
De Roeck 2019	9 mos	N/A	[32]
Chang 2023	5 mos	N/A	[33]
Mohandes 2020	until discharge	N/A	[34]
Kaczmarek 2021	10 mos	1 DRT, 1 PDL, 1 death	[35]
Marcon 2023	6 mos	N/A	[36]
Cruz-Gonzalez 2017	until discharge	N/A	[37]
Cammalleri 2016	until discharge	N/A	[38]
Pak 2013	1.5 mos	N/A	[39]
Yadav 2017	1.5 mos	N/A	[40]
Martins 2018	1 mo	N/A	[41]
Del Furia 2017	until discharge	N/A	[42]

*DRT* device-related thrombus, *PDL* peri-device leakage

N/A indicates data not available. Adverse clinical outcomes included a composite of death, stroke or other systemic thromboembolism, DRT, major bleeding, PDL, transient ischemic attack and device-related complications during follow-up. Major bleeding was defined as gastrointestinal bleeding, cerebral hemorrhage, intramuscular bleeding, etc.

## Discussion

**Fig. 4:**
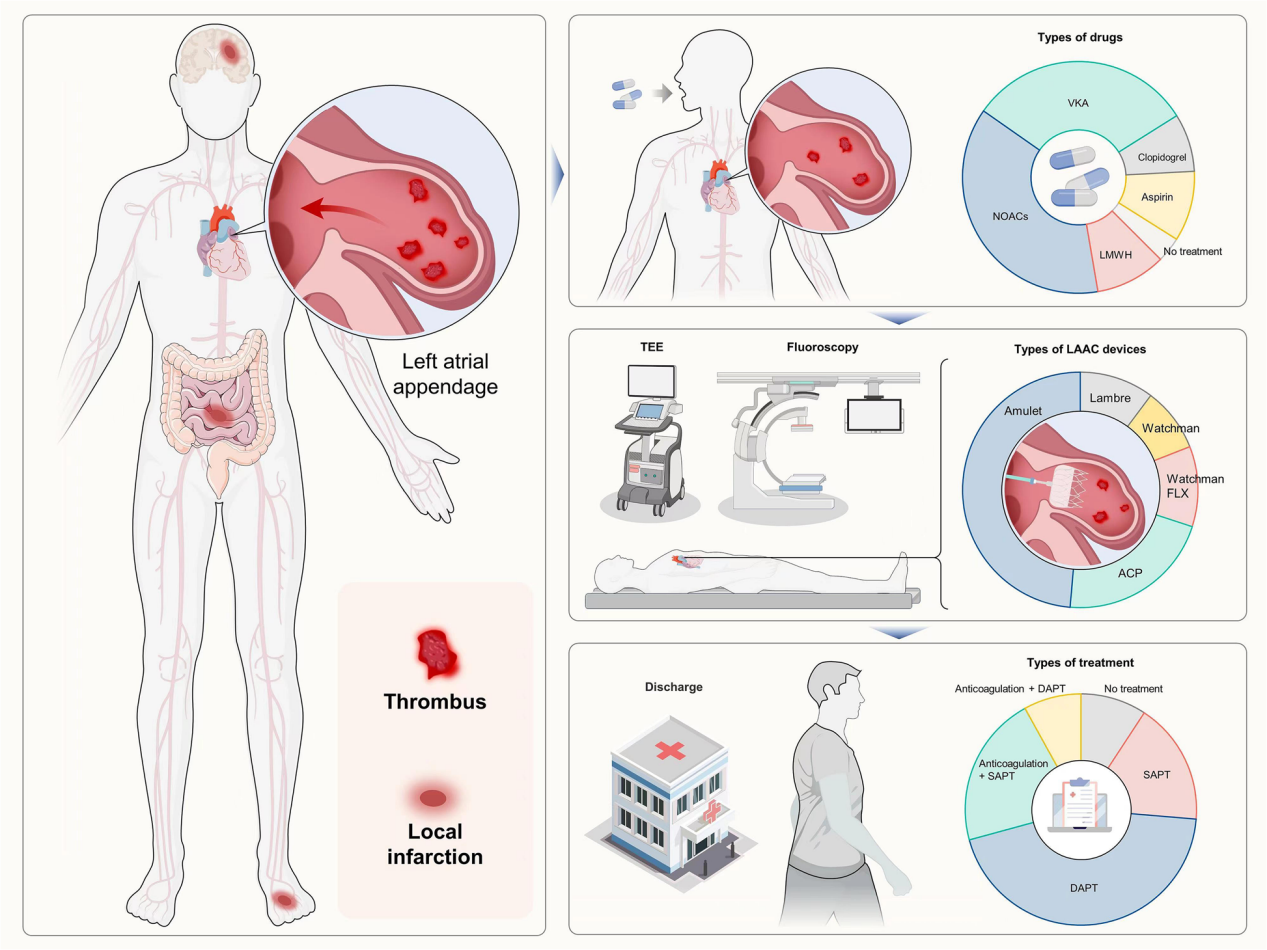
Graphical abstract for LAAC in the treatment of LAAT. ACP, Amplatzer cardiac plug; DAPT, dual antiplatelet therapy; LAAC, left atrial appendage closure; LAAT, left atrial appendage thrombus; LMWH, low-molecular-weight heparin; NOACs, novel oral anticoagulants; SAPT, single antiplatelet therapy; TEE, transesophageal echocardiography; VKA, vitamin K antagonist

This is a comprehensive systematic review of publications detailing LAAC procedures in patients presenting with LAAT. The main findings of this study were as follows: (i) The Amulet Amplatzer is currently the most commonly used LAAC device in patients with LAATs. (ii) Preprocedural OAC therapy and postprocedural DAPT are the main anticoagulation strategies for LAAT patients undergoing LAAC procedures, as these patients have a low rate of postprocedural stroke and DRT. (iii) The use of a no-touch technique, avoiding additional probing within the LAA, contributes to the safety of LAAC procedures. (iv) PDL is the most common adverse clinical outcome after LAAC procedures in LAAT patients; however, the overall incidence of adverse events is low. (v) While the combination of CPD with the LAAC procedure is associated with a low incidence of postprocedural stroke, the currently widespread implementation of CPD remains limited. In conclusion, the LAAC procedure is associated with preliminary effectiveness and safety in patients with persistent LAATs (Fig. 4).

AF is associated with a 4- to fivefold increased risk of ischemic stroke. The annual stroke risk in patients with AF ranges from 0.5% to 9.3%, and approximately 15% of ischemic strokes are attributed to AF [43, 44]. Chronic AF often remains asymptomatic and can go undetected in clinical practice, resulting in an underestimation of stroke risk. OAC therapy is regarded as the cornerstone for stroke prevention in patients with AF, and these agents are commonly prescribed prior to the LAAC procedure. To date, the specific treatment effect of NOACs on the formation of intracardiac thrombi has not been extensively investigated in large-scale randomized controlled trials (RCTs). However, preliminary evidence from case series and reports suggests that NOACs may represent a safe and effective option for treating intracardiac thrombus, particularly in cases where warfarin has been shown to be ineffective [45]. Furthermore, Nelles et al. [46] conducted a study that demonstrated LAAT resolution rates comparable between warfarin and NOACs, with NOACs resulting in a shorter time to thrombus resolution. Nevertheless, it is crucial to acknowledge that OACs cannot entirely eliminate LAAT [19]. For patients with persistent LAAT despite OAC therapy or those with contraindications to OAC therapy, the LAAC procedure emerges as a potential and viable alternative. Traditionally, LAAT has been considered a contraindication for LAAC. However, studies by Sharma et al. [14] indicate that LAAC may be a feasible option for patients with LAAT. By challenging traditional contraindications, LAAC offers additional treatment options for patients with persistent LAAT.

Procedural embolization is a severe complication that can occur during LAAC in patients with LAAT. Catheter manipulation within the LAA can potentially dislodge or detach the LAAT, leading to stroke or peripheral embolization events. For patients with LAATs, the combination of the no-touch technique can greatly enhance the safety of the procedure in addition to standard deployment techniques [20]. The no-touch technique involves minimizing guidewire or catheter manipulation within the LAA by loading the closure device with the delivery sheath in the left superior pulmonary vein [19]. Lange et al. [21] proposed using transesophageal echocardiography (TEE) to measure the diameter of the partially opened occluder and compared it to the size of the middle part of the LAA. The release of the device was continued until the diameter of the opened corpus of the occluder was greater than the measured value. This approach helps prevent deeper protrusion of the device into the LAA and reduces the risk of procedure-related thrombus dislodgement. While the "no-touch" technique has demonstrated encouraging outcomes in certain patients, its intricate procedure requires a sophisticated understanding of the LAA anatomy and highly skilled maneuvering of the devices. This complex procedure restricts its wide implementation, making it seemingly impractical for novice operators. Despite the promise of reduced complications and increased closure efficacy, the technical difficulty lies in delicate navigation and precise device placement within the complex and highly variable anatomy of the LAA.

CPD was originally proposed and developed in transcatheter aortic valve replacement (TAVR) and has been linked to a notable reduction in cerebral ischemic burden [47]. Although the PROTECTED TAVR study [48] revealed that the use of the Sentinel device (the first TAVR intraoperative CPD approved by the United States Food and Drug Administration) did not decrease the incidence of clinical stroke during the TAVR periprocedural period, it did report a decreased occurrence of disabling strokes. Recent prospective studies and case reports have indicated that the combined use of LAAC and cerebral protection systems is a safe and effective treatment option for patients with LAAT [22, 49, 50]. It is important to highlight that the majority of the current research involved organized and securely attached LAATs within the fundus of the LAA. This finding underlines a key limitation in the current literature and practice. Significantly, even with the use of CPDs, an LAAT protruding out of the LAA remains a strict contraindication for LAAC due to the high risk associated with its potential mobility. Furthermore, we address complex scenarios involving thrombi located at the neck of the LAA. When these thrombi exhibit any degree of mobility, they present a considerable challenge, leading most interventional cardiologists to prudently avoid attempting LAAC in such cases. In the future, large-scale RCTs investigating the use of LAAC in combination with cerebral protection systems for stroke prevention in LAAT patients may provide further clinical evidence.

Cardiac imaging plays a crucial role in LAAC procedures. The data presented in this systematic review indicate that TEE is the most frequently utilized modality for procedural imaging. TEE is considered the gold standard for diagnosing LAATs [51], with a positive predictive value of 100%, a negative predictive value of 98.9%, and a diagnostic accuracy of 99.1% [52]. Traditional 2D TEE has limitations in accurately assessing LAA function. Real-time 3D TEE is a valuable tool that minimizes artifact interference and enables a more precise analysis of the association between LAA functional parameters and LAAT [53]. However, 3D technology is limited by its lower spatial and temporal resolution than 2D TEE [54]. Intracardiac echocardiography (ICE) is an efficient alternative to TEE for visualizing cardiac structures [55]. A study conducted by Nielsen-Kudsk et al. demonstrated the successful utilization of ICE as a guide for LAAC with the Watchman FLX device. The study reported excellent procedural success, a high rate of effective LAAC, and minimal periprocedural complications [56]. In a porcine model, both ICE and TEE demonstrated similar imaging capabilities for visualizing LAAT. However, in patients with AF, ICE imaging showed lower sensitivity in detecting LAAT than did TEE [57, 58]. Considering the potential interaction between ICE and the LAAC sheath during the procedure and its typical supplementation with LAA angiography [14], the use of ICE in patients with LAAT should be performed by experienced operators who have conducted a minimum of 20 LAAC procedures per year.

## Limitations

This study has several limitations. 1) The retrospective design of this study introduces inherent known and unknown selection biases. Additionally, publication bias and outcome reporting bias may significantly influence the conclusions of our review. 2) The purpose of this study was primarily to assess the feasibility of LAAC in patients with LAAT. The applicability of these findings may be limited to experienced operators. 3) A notable limitation is the lack of detailed descriptions of LAAT characteristics in the included reports. The size, location, morphology, or mobility of LAATs significantly impacts the implantation and efficacy of LAAC devices. This gap underscores the need for more detailed investigations into LAAT characteristics in future RCTs. 4) The absence of long-term follow-up data in our study limits the ability to assess the extended-term effectiveness and safety of LAAC in patients with LAATs.

## Future directions

Targeted investigations are crucial for advancing the understanding of LAAC in patients with LAAT. Subsequent research should delve into detailed analyses of LAAT characteristics, including size or location, aiming to enhance procedural considerations. Long-term follow-up studies are needed to evaluate the efficacy of LAAC therapy. Moreover, there is a pressing need for comparative studies among different LAAC devices and well-designed RCTs to establish a higher level of evidence, offering clarity on the optimal approach for patients with LAATs. These future directions are geared toward refining clinical strategies and contributing to evidence-based decision-making in this specific patient population.

## Conclusion

In conclusion, this comprehensive systematic review elucidates the prospects of LAAC procedures in patients with LAATs. The Amulet Amplatzer is the most commonly used LAAC device in LAAT patients, and it achieves procedural effectiveness and safety through the combination of preprocedural OAC therapy and postprocedural DAPT. The no-touch technique has emerged as a crucial measure for enhancing the overall safety of LAAC procedures. Despite PDL being the primary adverse outcome, the overall incidence of adverse events remains low. Additionally, the incidence of postprocedural stroke is lower in LAAT patients with implanted CPDs, and CPD implementation is not widespread in this patient population. Our findings underscore the potential utility of LAAC in patients with LAAT. Future large-scale RCTs with long-term follow-up focusing on different LAAT characteristics and various LAAC device types may provide higher-quality clinical evidence for patients, guiding evidence-based decision-making in clinical practice.

### Supplementary Information

**Supplementary Material 1. **

## Data Availability

The datasets used and/or analysed during the current study available from the corresponding author on reasonable request.

## Abbreviations

AF: Atrial fibrillation
ACP: Amplatzer cardiac plug
CPD: Cerebral protection device
DAPT: Dual antiplatelet therapy
DRT: Device-related thrombus
ICE: Intracardiac echocardiography
LAA: Left atrial appendage
LAAC: Left atrial appendage closure
LAAT: Left atrial appendage thrombus
LMWH: Low-molecular-weight heparin
LSPV: Left superior pulmonary vein
NOACs: Novel oral anticoagulants
OAC: Oral anticoagulant
PDL: Peri-device leakage
RCTs: Randomized controlled trials
SAPT: Single antiplatelet therapy
TAVR: Transcatheter aortic valve replacement
TEE: Transesophageal echocardiography
